# Incorporation of protein binding effects into likelihood ratio test for exome sequencing data

**DOI:** 10.1186/s12919-016-0043-8

**Published:** 2016-10-18

**Authors:** Dongni Zhang, Hongzhu Cui, Dmitry Korkin, Zheyang Wu

**Affiliations:** 1 Mathematics Department, Worcester Polytechnic Institute, 100 Institute Road, Worcester, MA 01609-2280 USA; 2 Computer Science Department, Bioinformatics and Computational Biology Program, Worcester Polytechnic Institute, 100 Institute Road, Worcester, MA 01609-2280 USA

## Abstract

Statistical association studies are an important tool in detecting novel disease genes. However, for sequencing data, association studies confront the challenge of low power because of relatively small data sample size and rare variants. Incorporating biological information that reflects disease mechanism is likely to strengthen the association evidence of disease genes, and thus increase the power of association studies. In this paper, we annotate non-synonymous single-nucleotide variants according to protein binding sites (BSs) by using a more accurate BS prediction method. We then incorporate this information into association study through a statistical framework of likelihood ratio test (LRT) based on weighted burden score of single-nucleotide variants (SNVs). The strategy is applied to Genetic Analysis Workshop 19 exome-sequencing data for detecting novel genes associated to hypotension. The SNV-weighting LRT idea is empirically verified by the simulated phenotypes (336 cases and 1607 controls), and the weights based on BS annotation are applied to the real phenotypes (394 cases and 1457 controls). Such strategy of weighting the prior information on protein functional sites is shown to be superior to the unweighted LRT and serves as a good complement to the existing association tests. Several putative genes are reported; some of them are functionally related to hypertension according to the previous evidence in the literature.

## Background

Statistical association studies serve an important role in finding putative disease genes for further biological validation and reasoning. There are many association methods, but the common essential idea is to test the strength of statistical evidence for nonrandom variant distribution in cases and in controls. Such statistical evidence won’t be strong or reliable if data sample size is relatively small and/or single-nucleotide variant (SNV) mutations are rare, often the challenges imposed in current sequencing studies [1]. Furthermore, the mechanism of genetic effect is complex, not just a straight line from DNA to disease. A gene may be critical to a disease pathway, but the final disease status are affected by many other factors so that the association evidence measured solely by genotype and phenotype data could be weak. The most recent gene-hunting researches are challenged to find subtle disease genes responsible for the missing heritability, especially after the low-hanging fruits have been picked [2].

A promising approach to increasing the statistical power of association studies is to properly integrate the SNV information that reflects the intermediate steps of disease development. Protein–protein interactions (PPIs) are one important component related to disease development. Genetic factors may function through influencing PPIs. Several recent genome-wide association studies have reported the value of incorporating PPI information into the pipeline of identifying novel genes of type 1 diabetes and kidney dysfunction [3, 4]. However, their methodology mainly uses generic functional information, for example, Gene Ontology (GO) terms, to filter candidate genes and SNVs for test, while the association test itself is still traditional without implicitly incorporating such information [5]. Such filtering process can loosen the strict genome-wide significance level in favor of relatively weak association factors. However, it would be nicer to drop as few genes as possible, and at the same time to combine the prior biological information in a quantitative fashion.

In this study, we develop an association test framework that enables implicit incorporation of the prior importance of SNVs regarding their influence to PPI that may involve the intermediate steps of disease development. The basic idea is to group-test SNVs in functional units of associations, for example, genes, where SNV genotype values are weighted and thus prioritized according to their influence information. Our framework has two major components. First, proper generation of SNV weights based on the annotation of their effects on protein binding. Second, construction of a likelihood ratio test (LRT) to incorporate the SNV weights. The LRT has two advantages. First, the LRT statistic is essentially the ratio of the likelihoods between the null and the alternative regarding whether the distribution in cases differs from that in controls. Thus the LRT has enough flexibility to enable construction and incorporation of informative weights based on the meanings of the null and the alternative hypotheses. Second, the LRT is optimal for detecting weak and sparse signals [6, 7]. There are different versions of the LRT; we adapt a formulation by Chen et al. [8] as the prototype statistic for case-control studies.

## Methods

### Annotate non-synonymous single-nucleotide variants according to protein binding sites

The annotation pipeline relies on two in-house de novo binding-site prediction methods; the first is based on sequence, and the second is based on structure. For the sequence-based method, 4 Random Forrest classifiers are designed according to (a) the training sets the classifiers use (one for hetero-oligomeric structures and the other for homo-oligomers), and (b) the set of features calculated for each protein sequence. We use the method for predicting the hetero-oligomeric interaction binding sites. After training, each classifier predicts whether or not a residue in a protein sequence belongs to a protein-binding site. To generate the sets of features, we use a sequence-based sliding window of 9 consecutive residues, with the center residue (position 5) as the target. One input feature vector includes residue labels; each label corresponds to 1of the 9 positions of a sequential sliding window [9]. A second sequence-based feature vector includes the sequence length and the distances between the target residue and the closest 20 essential residues. The classifiers are implemented using Scikit-learn libraries for Random Forest [10]. For all classifiers, the number of the Decision Trees parameter is set to 200. The other parameters are set at the library’s default.

Both sequence-based and structure-based methods have been assessed using 10-fold cross-validation on a set of 335 protein sequences with their binding sites extracted from hetero-oligomeric complexes from the DOMMINO (Database of Macromolecular Interactions) database [11]. We then select a classifier with the highest f-measure value defined by, where *Pr* and *Re* are the precision and recall values, respectively. Our best-performing sequence-based classifier for hetero-oligomeric interactions reports an f-measure of 0.38 and an accuracy of 64 %, outperforming the state-of-the-art approach PSIVER (a server for the prediction of protein–protein interaction sites in protein sequences) (f = 0.36, accuracy = 60 % on the same set) [12].

Similarly, for the structure-based method, 4 Random Forrest classifiers are designed according to (a) the training sets (hetero-oligomers vs. homo-oligomers), and (b) the type of the sliding window (sequence/structure) for generating features. The input feature vector includes (1) labels of 9 residues in the sliding window, (2) secondary structure of the center residue, (3) average hydrophobicity, (4) statistics on the residue accessible surface area (ASA), (5) average depth index (DPX), (6) statistics on protrusion, and (7) length of the sequence. The secondary structure is from DSSP (dictionary of protein secondary structure) [13] and other features are extracted from PSAIA (protein structure and interaction analyzer) [14]. As in the case of sequence-based classifiers, we apply a heterodimer structure-based binding site classifier with the highest f-measure obtained during 10-fold cross-validation on the same data set. The best-performing structure-based classifier achieves an f-measure of 0.46 and an accuracy of 73 %, also outperforming the state-of-the-art approach PINUP (protein interface residue prediction) (f = 0.33, accuracy = 63 % on the same set) [15].

The variant data provided in the VCF (variant call format) files of the odd numbers of chromosomes for uncorrelated individuals are preprocessed using ANNOVAR (Annotate Variation) to retrieve SNV locations on the genes [16]. One of the current bottlenecks of the binding-site prediction methods is the computational complexity of obtaining the features, especially for the structure-based approach. As a result, for this proof-of-concept paper we have applied the sequence- and structure-based approaches to the data obtained in the first sample, covering 4457 non-synonymous single-nucleotide variants (nsSNVs) on 2711 genes regarding to whether they are located on protein binding sites or not. A large-scale all-sample analysis is estimated to require approximately 20,000 CPU hours of computation and will follow.

### Likelihood ratio tests

Consider group-testing SNVs in genes as functional units. For the $j$ th gene, the generic LRT formula for the case-control study is

1 $$ \Lambda \left({g}_j\right)= \log \left({L}^A{L}^U/L\right) $$

where ${L}^A$, ${L}^U$, and $L$ are the likelihoods for the distributions of an appropriate disease-association measure in cases, in controls, and in both groups, respectively. The numerator of the LRT separates the likelihoods in cases and in controls to model the alternative hypothesis that there exists an association in terms of the likelihood differentiation between the two groups; the denominator pools the data of cases and controls together for the likelihood of the null hypothesis of no association. Here we adapt an LRT based on Bernoulli likelihoods [8]:

2 $$ \Lambda \left({g}_j\right)= \log \frac{{\left({\widehat{p}}_j^A\right)}^{T_j^A}{\left(1-{\widehat{p}}_j^A\right)}^{\left(m-{T}_j^A\right)}{\left({\widehat{p}}_j^U\right)}^{T_j^U}{\left(1-{\widehat{p}}_j^U\right)}^{\left(l-{T}_j^U\right)}}{{\left({\widehat{p}}_j\right)}^{T_j}{\left(1-{\widehat{p}}_j\right)}^{\left(m+l-{T}_j\right)}} $$

where $m$ and $l$ are the number of cases and controls, and ${T}_j^A$, ${T}_j^U$ and ${T}_j$ (${\widehat{p}}_j^A$, ${\widehat{p}}_j^U$ and ${\widehat{p}}_j$) are the total numbers (and the corresponding estimated proportions) of the burden scores that exceed a threshold $t$ in cases, controls, and both groups, respectively.

The burden scores are the collapsed genotype values over variants on a gene, which measure the overall variant distributions [17]. Specifically, for the $j$ th gene of the individual $k$, the burden score is

3 $$ {S}_{jk}={\displaystyle {\sum}_{i=1}^{n_j}{x}_{ik}} $$

where ${x}_{ik}$ is the genotype value (0, 1, or 2 according to the copy number of the minor allele) of SNV $i$ of individual $k$. We search a sequence of threshold $t$ and choose the value that maximizes the test statistic. LRT tests the unevenness of burden scores in cases versus controls, not just their enrichment in cases. So, when ${\widehat{p}}_j^A$ ≤ ${\widehat{p}}_j^U$, the test statistic is adjusted to be

4 $$ \Lambda \left({g}_j\right)= \log \frac{{\left({\widehat{p}}_j^U\right)}^{T_j^A}{\left(1-{\widehat{p}}_j^U\right)}^{\left(m-{T}_j^A\right)}{\left({\widehat{p}}_j^A\right)}^{T_j^U}{\left(1-{\widehat{p}}_j^A\right)}^{\left(l-{T}_j^U\right)}}{{\left({\widehat{p}}_j\right)}^{T_j}{\left(1-{\widehat{p}}_j\right)}^{\left(m+l-{T}_j\right)}} $$

A gene-specific permutation test is applied to calculate *p* values. It provides a proper control of the type I error rate by accommodating the heterogenous gene sizes and minor allele frequencies, the dependence among SNVs, and other potential departure from Bernoulli model assumptions in real data.

### Likelihood ratio test with burden scores weighted by the effect direction

The above LRT tests in equations (2) and (4) consider the directionality of the burden scores in equation (3). However, because SNV genotypes have directionality, there could exist both protective and deleterious SNVs that have reversed variant distributions in cases and in controls. The uneven distributions would disappear in burden scores after summing their genotype values. To avoid such cancellation of the opposite genetic effects, we consider switching all potential effect direction consistently before calculating the burden score. Equivalently, we can weigh the burden score:

5 $$ {S}_{jk}={\displaystyle {\sum}_{i=1}^{n_j}{s}_i{x}_{ik}} $$

where ${s}_i=1$ if ${\widehat{p}}_j^A$ ≤ ${\widehat{p}}_j^U$ and ${s}_i= - 1$ otherwise. To properly control the type I error, such direction weight is recalculated in each loop of the permutation test.

### Likelihood ratio test with burden scores weighted by protein-binding-site information

The LRT with burden scores weighted by protein-binding-site information (LRT-BS) is similar to the LRT with burden scores weighted by the effect direction (LRT-DIR) in equation (5) except that ${s}_i$ are the weights based on the importance of variants in terms of whether they are located on protein-binding sites. A key issue here is to decide the principle for assigning values to ${s}_i$. To address this issue, we take advantage of the simulation data, where we know the genotype–phenotype map. By comparing the statistical power, we can systematically evaluate a spectrum of strategies with various magnitudes and both directions of the weights. Guidance can be drawn from a robust weighting strategy that is good for various types of genetic effects: protective, deleterious, and weak effects. One practice of this idea is described in “Finding a weighting principle for binding site annotation” below.

### Combined multivariate and collapsing and C-alpha methods

We compared the LRT-based methods with two widely applied association tests for sequencing data. The Combined Multivariate and Collapsing (CMC) method [18] is a classic collapsing strategy. Similar to the burden score–based tests, it evidences the genetic association through how unevenly the mean allele frequencies are distributed in cases and in controls. In contrast, the C-alpha method [19] tests the variance of allele frequencies. Either method is a typical association test strategy used here to compare with the LRT methods proposed here.

### Implementation

Regarding the implementation of the above methods, the software Variant Tools [20, 21] was applied to manipulate data, to carry out the C-alpha and CMC methods, and to call our own R functions, which were being implemented for the LRT-related methods. To ease the computation demand, we adopted a 2-stage permutation strategy: a permutation loop continues into the second stage only if the concurrent empirical *p* value is less than 0.1.

## Results

### Finding a weighting principle for binding site annotation

**Fig. 1:**
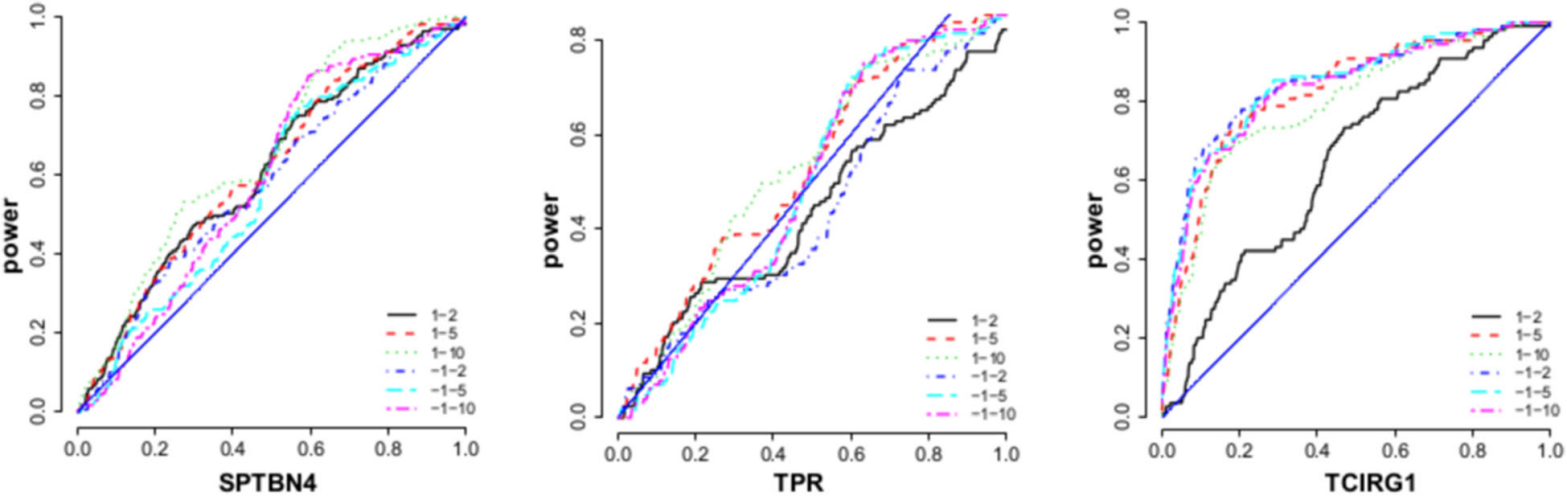
Statistical power under various weighting schemes. Three genes from the simulation answer sheet: *SPTBN4*, *TPR*, and *TCIRG1*. Six combinations of weights for the first 10 functional variants (2, 5, or 10) and for the rest variants (1 or −1)

Based on the known genotype-phenotype map in simulation data, we systematically evaluated various strategies of weighting SNVs. We used genotype data of the exome sequencing variants on odd numbers of chromosomes for uncorrelated individuals; the phenotypes were the hypertension status of the 200 simulations (336 cases and 1607 controls). We chose 3 genes with various types of genetic effects: *SPTBN4* (containing functional SNVs with relatively strong protective effects), *TPR* (containing functional SNVs with relatively weak effects), and *TCIRG1* (containing functional SNVs with relatively strong deleterious effects). To mimic the reality that the annotation of functional SNVs is often incomplete, we arbitrarily chose the first 10 functional SNVs (according to their positions) as annotated, and treated the rest of the functional SNVs in the same way as the nonfunctional SNVs. So that we could systematically consider a variety of strategies with various magnitudes and the relative directionality of weighting, we studied 6 combinations of the following weights: the 10 functional SNVs were assigned ${s}_i$ = 2, 5 or 10, and the rest of the SNVs were assigned ${s}_i$ = 1 or −1. The power curves in Fig. 1 show that different weighting schemes perform differently for genes with different genetic mechanisms. However, the combination 1 and 10 has a robust performance overall. It seems a good principle to assign functional SNVs and the rest with weights that have the same direction (i.e., the same sign) but relatively large difference in magnitude. Thus, for applying LRT-BS in real data analysis, we assigned weight ${s}_i$ = 10 for nsSNVs in a binding site, 5 for nsSNVs not in a binding site, and 1 for the rest.

### Real data analysis

**Fig. 2:**
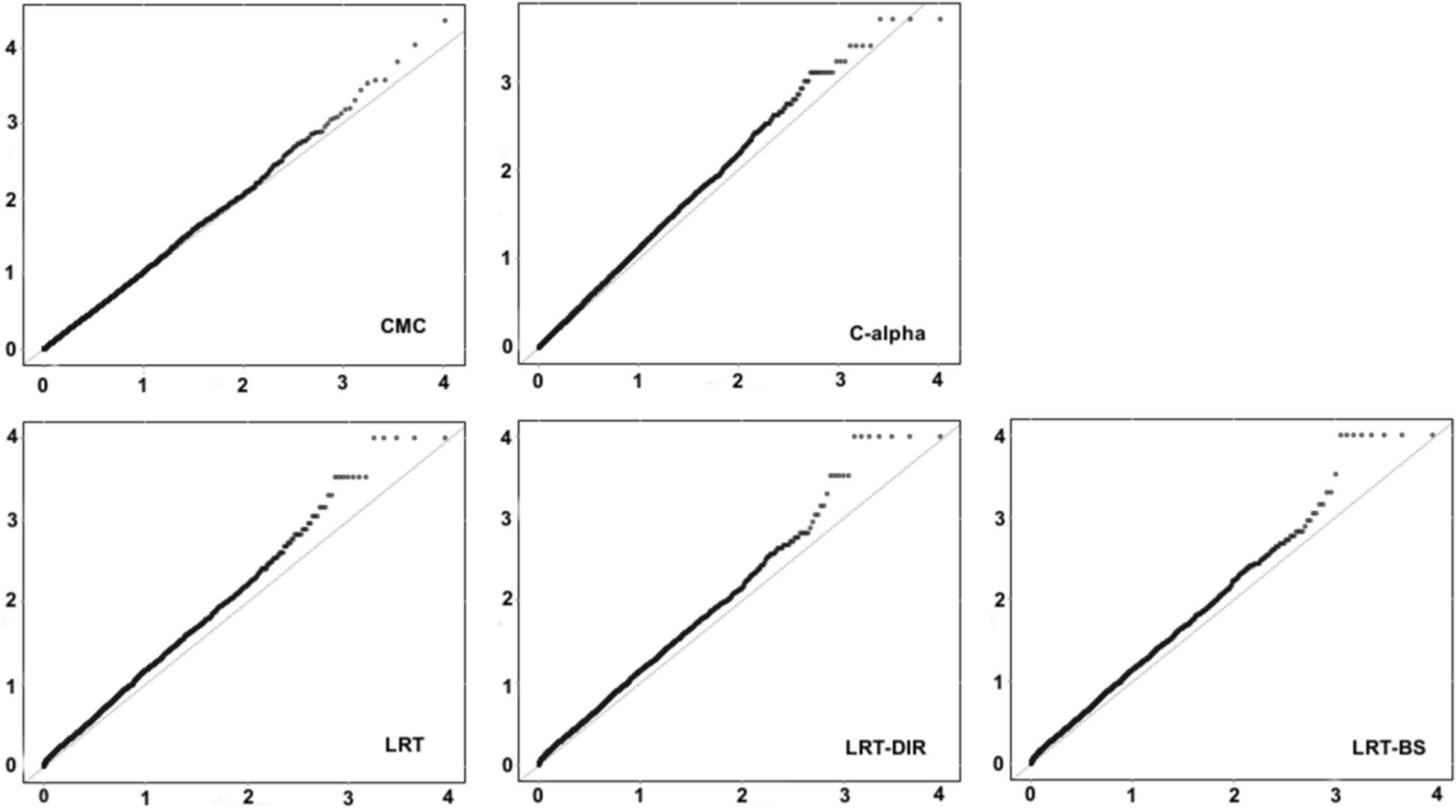
Quantile–quantile (Q-Q) plots of 4 association tests for real data analysis. Upper right corner dots represent the top-ranked genes: 4 genes for C-alpha(genomic inflation factor λ=1.11); 3 genes for CMC (λ=1.03); 5 genes for LRT(λ=1.27);7 genes for LRT-DIR (λ=1.28);and 8 genes for LRT-BS (λ=1.28). The *x*-axis is $-{log}_{10}{P}_{expected}$; the *y*-axis is $-{log}_{10}{P}_{observed}$

Based on the real diastolic blood pressure (DBP) and systolic blood pressure (SBP) observations, hypertension cases were defined as individuals with SBP greater than or equal to 140 or DBP greater than or equal to 90 according to the American Heart Association. This cutoff led to 394 cases and 1457 controls. Five methods were applied to testing the association of genes annotated by National Center for Biotechnology Information (NCBI) RefSeqGene database: C-alpha test, CMC test, LRT, LRT-DIR, and LRT-BS. Figure 2 shows the quantile–quantile (Q-Q) plots of the resulting *p* values. CMC and C-alpha methods gave better (lower) genomic inflation factors (λ) than LRT methods. However, more important than the *p* values themselves is their ordering profile. An association test is good if its ordered *p* values lead to the genes being ordered according to their true relevance to the disease. Such consistency is the essence of providing high power and a lower false discovery rate. A slightly higher genomic inflation factor only means that we need a *p* value cutoff slightly lower than the nominal threshold. Based on this consideration, here we compare different methods according to their top-ranked genes that are obviously standing out. These genes are illustrated by the dots on the upper right corners of the Q-Q plots in Fig. 2 at a somewhat arbitrary *p* value cutoff of 1.55E-4. The C-alpha test yielded 4 genes, CMC yielded 3 genes, and LRT, LRT-DIR, and LRT-BS yielded 5, 7, and 8 genes, respectively. The three LRTs have the same level of λ, indicating the same capacity of controlling the type I error. Because LRT-DIR and LRT-BS have more genes discovered than LRT, they are likely more powerful.

Table 1 summarizes the top genes yielded by the 5 methods. Searching the literature shows that many of the genes are related to hypertension. In particular, *ABO* has been reported as protecting against hypertension in a Spanish population [22], and is related to ischemic heart disease in a Pakistani population [23]. *MSL2*is located at 135,867,760 to 135,916,083 bp on chr3, which locates inside of *BP24_H*, the blood pressure quantitative trait locus (QTL) #24 (human) at 135,438,224 to 161,438,224 bp. *ITGA2* is reported to be associated with hypertension in a Japanese population [24]. *PSPC1*is reported to be related to hypertension directly [25]. *TLN2* is related to pulmonary arterial hypertension (PAH) [26]. *ZNF557* features the*GGA28-*related chromosome rearrangements in primate genome, while the *GGA28* variation is associated with pulmonary hypertension syndrome [27]. *ZMYM5* is related to lung hypertension recovery (U74Cv2) according to the Gene Expression Omnibus (GEO) profiles. *PAEP* is reported to be downregulated for preeclampsia, which is defined based on hypertension [28]. Increases in *BCHE* activity may be observed in patients with nephrotic syndrome, which is related to hypertension [29]. The location of *ZBTB4* (7,362,685 to 7,387,582 bp) is inside of *BP15_H* (the blood pressure QTL#15[human]) on chr17. *DNAH9* gene cluster is reported to be related to young-onset hypertension [30]. Through common microRNAs, *LUC7L2* was found to be associated with hydrochlorothiazide (HCTZ)-induced uric acid elevations in an antihypertensive responses study of African Americans [31].

**Table 1:** *P* Values of the top genes based on analyzing real data

Genes	Chr	C-alpha (4 genes)	CMC (3 genes)	LRT (5 genes)	LRT-DIR (7 genes)	LRT-BS (8 genes)
*C7orf55-LUC7L2*	7	1.99E-04 (1)	9.80E-03 (115)	2.79E-01 (3005)	2.41E-01 (2659)	2.13E-01 (2385)
***LUC7L2***	7	1.99E-04 (1)	9.80E-03 (115)	2.57E-01 (2773)	2.85E-01 (3119)	2.25E-01 (2496)
***DNAH9***	17	1.99E-04 (1)	4.11E-02 (495)	8.81E-01 (8461)	9.25E-01 (8834)	7.53E-01 (7822)
*AKAP8*	19	1.99E-04 (1)	4.21E-02 (504)	2.43E-01 (2636)	2.29E-01 (2534)	2.75E-01 (3021)
***MSL2***	3	3.80E-3 (68)	4.39E-05 (1)	2.00E-01 (2)	≤1.00E-4 (1)	≤1.00E-4 (1)
***ZBTB4***	17	8.00E-4 (12)	9.21E-05 (2)	1.83E-02 (246)	4.11E-02 (525)	2.35E-01 (2479)
***BCHE***	3	1.86E-2 (287)	1.55E-04 (3)	9.11E-2 (1117)	8.53E-2 (1070)	8.69E-2 (1073)
*LZIC*	1	7.20E-3 (112)	1.00E-2 (121)	≤1.00E-4 (1)	≤1.00E-4 (1)	≤1.00E-4 (1)
***ABO***	9	4.08E-1 (4715)	1.92E-2 (2112)	6.00E-4 (4)	≤1.00E-4 (1)	4.00E-4 (3)
*COL15A1*	9	7.36E-1 (7836)	1.56E-1 (2030)	≤1.00E-4 (1)	≤1.00E-4 (1)	≤1.00E-4 (1)
***PSPC1***	13	3.06E-1 (3366)	6.73E-1 (7481)	2.00E-4 (2)	≤1.00E-4 (1)	≤1.00E-4 (1)
***TLN2***	15	3.70E-1 (4036)	3.63E-1 (4173)	2.00E-4 (2)	≤1.00E-4 (1)	≤1.00E-4 (1)
***ZNF557***	19	6.45E-1 (6876)	2.38E-1 (2870)	4.00E-4 (3)	≤1.00E-4 (1)	≤1.00E-4 (1)
*ITGA2*	5	6.69E-1 (7123)	5.08E-1 (5776)	≤1.00E-4 (1)	2.00E-4 (2)	4.00E-4 (3)
***ZMYM5***	13	8.51E-1 (8932)	7.67E-1 (9624)	2.00E-4 (2)	2.00E-4 (2)	≤1.00E-4 (1)
*YIPF2*	19	4.18E-1 (4538)	6.24E-2 (1007)	≤1.00E-4 (1)	2.00E-4 (2)	≤1.00E-4 (1)
***PAEP***	9	4.52E-1 (4862)	4.92E-1 (5617)	≤1.00E-4 (1)	2.00E-3 (31)	6.00E-4 (4)

Bolded genes are found related to hypertension in literature. The underlined *p* values are less than 1.55E-5. Gene ranks are given in parentheses under the *p* values

## Discussion

The real data analysis shows that LRT-type methods are a good complementary strategy to traditional methods such as CMC and C-alpha. However, the genomic inflation factor is relatively high for the LRT-type methods. It is left for future research to better calculate their *p* values. Furthermore, the current method is designed to address the case-control association studies. Another future research project is to apply the LRT principle for quantitative traits. The key is to set up proper likelihood functions to model the connections between genotype and response under the null and the alternative.

The annotations for protein-binding sites are still limited in reflecting the genetic influence to the PPIs and disease mechanism. We are carrying out further computations to annotate the SNV effects regarding individual PPIs as well as the PPI network as a whole [32]. The annotation could provide more specific properties of the SNV effects, such as being characterized as beneficial, deleterious, or neural. Such annotations will provide more information on how nsSNVs are involved into the disease process, and thus could further improve the power of association tests. One advantage of LRT testing is that it can construct likelihood ratios for different sources of association measures and combine them into a single test statistic [8]. The computation load is relatively heavy because of the dependence on permutation test, but the real data analysis shows that it is affordable to use such a method for exome sequencing study at the gene level.

## Conclusions

Biological information on SNVs can be helpful to improve the association tests. We developed a framework to incorporate the protein-binding-site indicator of nsSNV into a LRT test, which has the similar genomic inflation factor as the original LRT test, but which provides more power to detect disease-associated genes. Through analyzing Genetic Analysis Workshop 19 exome-sequencing data on odd-numbered chromosomes, we discovered that many top-ranked genes are indeed related to hypertension, thus evidencing the effectiveness of this framework.
